# Predictors for development of denosumab-induced hypocalcaemia in cancer patients with bone metastases determined by ordered logistic regression analysis

**DOI:** 10.1038/s41598-020-80243-y

**Published:** 2021-01-13

**Authors:** Yuko Kanbayashi, Koichi Sakaguchi, Fumiya Hongo, Takeshi Ishikawa, Yusuke Tabuchi, Osamu Ukimura, Koichi Takayama, Tetsuya Taguchi

**Affiliations:** 1 Department of Outpatient Oncology Unit, University Hospital, Kyoto Prefectural University of Medicine, Kyoto, Japan; 2 Department of Education and Research Center for Clinical Pharmacy, Osaka University of Pharmaceutical Sciences, 4-20-1 Nasahara, Takatsuki, Osaka 569-1094 Japan; 3 Departments of Endocrine and Breast Surgery, Kyoto Prefectural University of Medicine, Kyoto, Japan; 4 Department of Urology, Kyoto Prefectural University of Medicine, Kyoto, Japan; 5 Department of Molecular Gastroenterology and Hepatology, Kyoto Prefectural University of Medicine, Kyoto, Japan; 6 Department of Pharmacy, University Hospital, Kyoto Prefectural University of Medicine, Kyoto, Japan; 7 Department of Pulmonary Medicine, Kyoto Prefectural University of Medicine, Kyoto, Japan

**Keywords:** Cancer, Epidemiology, Risk factors

## Abstract

This retrospective study was undertaken to identify predictors for the development of hypocalcaemia even with prophylactic administration of calcium and vitamin D, and to help guide future strategies to improve the safety, efficacy, and QOL of patients receiving denosumab. Between January 2016 and February 2020, a total of 327 advanced cancer patients at our hospital who were receiving denosumab were enrolled. Variables associated with the development of hypocalcaemia were extracted from the clinical records. The level of hypocalcaemia was evaluated using CTCAE version 5. Multivariate ordered logistic regression analysis was performed to identify predictors for the development of hypocalcaemia. Optimal cut off thresholds were determined using ROC analysis. Values of *P* < 0.05 (2-tailed) were considered significant. 54 patients have developed hypocalcemia (≥ Grade 1). Significant factors identified included concomitant use of vonoprazan [odds ratio (OR) = 3.74, 95% confidence interval (CI) 1.14–12.26; *P* = 0.030], dexamethasone (OR = 2.45, 95%CI 1.14–5.42; *P* = 0.022), pre-treatment levels of serum calcium (OR = 0.27, 95%CI 0.13–0.54; *P* < 0.001), ALP/100 (OR = 1.04, 95%CI 1.01–1.07; *P* = 0.003), and haemoglobin (OR = 0.79, 95%CI 0.68–0.93; *P* = 0.004). ROC curve analysis revealed that the threshold for pre-treatment levels of serum calcium was ≤ 9.3 mg/dL, ALP was ≥ 457 U/L, and haemoglobin was ≤ 10.4 g/dL. In conclusion, concomitant use of vonoprazan or dexamethasone, and pre-treatment levels of serum calcium (low), ALP (high) and haemoglobin (low) were identified as significant predictors for the development of denosumab-induced hypocalcaemia.

## Introduction

Denosumab is frequently used to prevent skeletal-related events in advanced cancer patients with bone metastasis^1,2^. On the other hand, serious hypocalcaemia has been reported as an adverse effect after the administration of denosumab. Therefore, to prevent hypocalcaemia, prophylactic administration of calcium and vitamin D is recommended concomitant with the administration of denosumab^3,4^. However, even with prophylactic administration, patients sometimes develop hypocalcaemia. Concomitant medications such as proton pump inhibitors (PPIs)^5–7^ and steroids^8^, and pre-treatment levels of serum calcium and alkaline phosphatase (ALP)^9,10^ have been reported as risk factors for hypocalcaemia, but their probability has not been determined. This retrospective study was thus undertaken to identify predictors associated with the development of denosumab-induced hypocalcaemia even with prophylactic administration of calcium and vitamin D, and to help guide future strategies to improve the safety, efficacy, and QoL of cancer patients with bone metastasis treated using denosumab.

## Results

**Figure 1:**
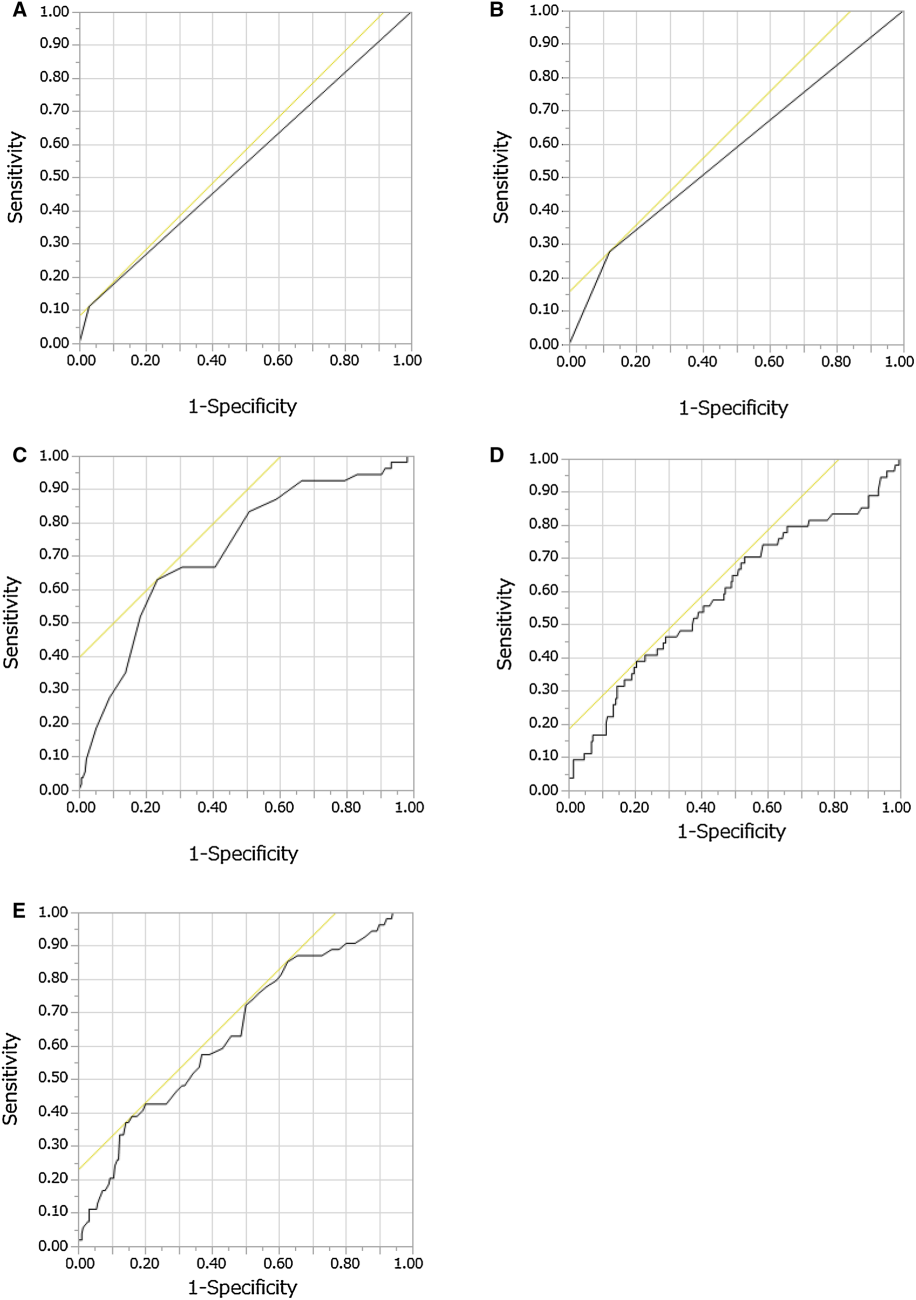
ROC curves about hypocalcaemia (≥ Grade 1) according the logistic regression significant variables. (**A**) ROC curve of concomitant use of vonoprazan with a sensitivity of 11.1% and specificity of 97.1% (AUC = 0.54). (**B**) ROC curve of dexamethasone with a sensitivity of 27.8% and specificity of 87.9% (AUC = 0.58). (**C**) ROC curve of the pre-treatment levels of serum calcium with a sensitivity of 63.0% and specificity of 76.6% (AUC = 0.72). (**D**) ROC curve of alkaline phosphatase with a sensitivity of 38.9% and specificity of 79.5% (AUC = 0.59). (**E**) ROC curve of haemoglobin with a sensitivity of 38.9% and specificity of 83.4% (AUC = 0.65).

Of the 348 patients who received denosumab, 21 were excluded from this study (no preventive medication for hypocalcaemia, n = 1; insufficient data, n = 20). Table 1 presents the clinical characteristics of the 327 enrolled patients, the potential variables related to the development of hypocalcaemia, and the results of univariate analyses. The forward stepwise selection procedure identified five variables (concomitant use of vonoprazan, dexamethasone, calcium level at the start of denosumab, ALP and haemoglobin). Multivariate ordered logistic regression analysis was performed using these variables. Significant factors identified for the development of hypocalcaemia included concomitant use of vonoprazan, dexamethasone, pre-treatment levels of serum calcium, ALP/100, and haemoglobin (Table 2). The ROC curve analysis of the group likely to develop hypocalcaemia (≥ Grade 1) revealed concomitant use of vonoprazan with a sensitivity of 11.1% and specificity of 97.1% (AUC = 0.54) (Fig. 1A), dexamethasone with a sensitivity of 27.8% and specificity of 87.9% (AUC = 0.58) (Fig. 1B). The ROC curve analysis revealed that the threshold for the calcium level at the start of denosumab was ≤ 9.3 mg/dL, with 63.0% sensitivity and 76.6% specificity [area under the curve (AUC) = 0.72] (Fig. 1C), ALP threshold was ≥ 457 U/L, with 38.9% sensitivity and 79.5% specificity (AUC = 0.59) (Fig. 1D), and haemoglobin threshold was ≤ 10.4 g/dL with 38.9% sensitivity and 83.4% specificity (AUC = 0.65) (Fig. 1E).

**Table 1:** Patient characteristics, extracted variables, and results of univariate analyses (n = 327).

	Grade 0 (n = 273)	Grade 1 (n = 50)	Grade 2 (n = 4)	*P* value	Odds ratio (95%CI)
**Demographic data**					
Male, n (%)	142 (52.0)	30 (60.0)	3 (75.0)	0.213	1.46 (0.81–2.65)
Age (year), median (range)	69 (23–89)	72 (38–92)	67.5 (60–71)	0.107	1.02 (1.00–1.05)
Height (cm), median (range)	161 (139–190)	160 (139–179)	166 (156–174)	0.812	1.00 (0.96–1.03)
Weight (kg), median (range)	55.0 (31.0–90.0)	54.0 (28.0–85.9)	54.5 (44.1–75.0)	0.393	0.99 (0.96–1.02)
BMI (kg/m^2^), median (range)	21.3 (13.5–35.8)	21.0 (14.1–27.2)	19.1 (18.1–26.6)	0.338	0.96 (0.89–1.04)
BSA (m^2^), median (range)	1.58 (1.13–2.08)	1.56 (1.07–2.05)	1.62 (1.40–1.85)	0.454	0.54 (0.11–2.74)
**Cancer type**					
Lung, n (%)	64 (23.4)	15 (30.0)	1 (25.0)	0.342	1.37 (0.72–2.61)
Breast, n (%)	101 (37.0)	9 (18.0)	1 (25.0)	0.011*	0.39 (0.19–0.81)
Urinary, n (%)	82 (30.0)	20 (40.0)	2 (50.0)	0.120	1.61 (0.88–2.93)
Prostate, n (%)	54 (19.8)	16 (32.0)	2 (50.0)	0.027*	2.05 (1.09–3.89)
Digestive, n (%)	25 (9.2)	5 (10.0)	0	0.994	1.00 (0.36–2.74)
Unknown, n (%)	1 (0.4)	1 (2.0)	0	–	–
**Comorbidity**					
Diabetes mellitus, n (%)	30 (11.0)	4 (8.0)	1 (25.0)	0.741	0.85 (0.32–2.27)
**Laboratory test value before administration**					
Serum creatinine, mg/dL, median (range)	0.71 (0.33–1.92)	0.71 (0.29–1.63)	0.62 (0.56–0.8)	0.161	0.41 (0.12–1.42)
Creatinine clearance, mL/min, median (range)	72.1 (17.7–217.3)	73.0 (18.8–153.4)	84.2 (68.8–108.8)	0.855	1.01 (1.00–1.01)
Creatinine clearance grade** (0/1/2/3)	148/39/80/6	28/7/12/3	3/1/0/0	0.762	0.95 (0.70–1.30)
Albumin, g/dL, median (range)	3.8 (1.7–5)	3.8 (1.9–4.5)	4.2 (3.8–5.1)	0.938	1.02 (0.64–1.61)
Alkaline phosphatase/100, U/L, median (range)	2.76 (0.71–34.29)	3.21 (0.77–7028)	6.49 (1.75–119.3)	< 0.001*	1.05*** (1.02–1.09)
Hemoglobin, g/dL, median (range)	12.4 (6.3–17)	11.5 (5.7–14.6)	12.6 (9.9–14.8)	< 0.001*	0.78 (0.67–0.90)
Calcium, mg/dL, median (range)	9.6 (8.3–14.7)	9.2 (8.3–12.7)	9.3 (8.9–9.6)	< 0.001*	0.27 (0.14–0.53)
**Number of cycles**	7 (1–91)	3 (1–68)	2 (1–42)	0.046*	0.97 (0.95–0.99)
**Concomitant medication**					
*Steroids, n (%)*	99 (36.3)	21 (16.9)	4 (100.0)	0.134	1.57 (0.87–2.82)
Prednisolone, n (%)	38 (13.9)	8 (16.0)	0	0.897	1.06 (0.46–2.42)
Dexamethasone, n (%)	33 (68.8)	12 (25.0)	3 (6.25)	0.002*	2.97 (1.49–5.42)
Betamethasone, n (%)	29 (10.6)	1 (2.0)	1 (25.0)	0.138	0.33 (0.08–1.42)
*Proton pump inhibitor, n (%)*	130 (47.6)	27 (54.0)	3 (75.0)	0.271	1.39 (0.77–2.50)
Esomeprazole, n (%)	61 (22.3)	8 (16.0)	0	0.210	0.60 (0.27–1.34)
Rabeprazole, n (%)	15 (5.5)	0	1 (25.0)	0.298	0.35 (0.05–2.54)
Lansoprazole, n (%)	42 (15.4)	12 (24.0)	2 (50.0)	0.052	1.97 (0.99–3.92)
Omeprazole, n (%)	4 (1.5)	1 (2.0)	0	0.847	1.24 (0.14–11.5)
Vonoprazan, n (%)	8 (2.9)	6 (12.0)	0	0.016*	3.85 (1.28–11.5)

CI, confidence interval; BMI, body mass index; BSA, body surface area.

*P < 0.05.

**Evaluated by National Cancer Institute Common Terminology Criteria for Adverse Events (NCI‑CTCAE) version 5.

***Odds ratio per ALP 100 U/L.

**Table 2:** Results of multivariate ordered logistic regression analysis for variables extracted by forward selection (n = 327).

Variable	*P* value	Odds ratio	95%CI	
			Lower 95%	Upper 95%
Dexamethasone	0.022*	2.45	1.14	5.42
Vonoprazan	0.030*	3.74	1.14	12.26
Calcium	< 0.001*	0.27	0.13	0.54
Haemoglobin	0.004*	0.79	0.68	0.93
Alkaline phosphatase/100	0.003*	1.04**	1.01	1.07
Lansoprazole	0.078	2.05	0.92	4.53

CI, confidence interval.

*P < 0.05.

**Odds ratio per ALP 100 U/L.

## Discussion

The multivariate ordered logistic regression analysis performed in this study showed that significant predictors for the development of denosumab-induced hypocalcaemia even when receiving prophylactic administration of calcium and vitamin D included concomitant use of vonoprazan and dexamethasone, and pre-treatment levels of serum calcium, ALP and haemoglobin. On ROC curve analysis of the potential factors responsible for the development of hypocalcaemia, the cut-off value for the calcium level at the start of denosumab was ≤ 9.3 mg/dL, that of ALP was ≥ 457 U/L, and that of haemoglobin was ≤ 10.4 g/dL.

For concomitant drugs, vonoprazan and dexamethasone were extracted as significant factors in this study. Previous research has reported that co-administration of PPIs was significantly related to hypocalcaemia^5–7^. Regarding the relationship between combined use of PPIs and the occurrence of hypocalcaemia, an increase in gastric pH due to the inhibitory effect of PPIs on gastric acid secretion has been reported to decrease the absorption of calcium, which becomes soluble under acidic conditions^5–7^. In this study, among the PPIs, only vonoprazan was extracted as a significant factor. Conventional PPIs are unstable in the acidic environment of the stomach and do not remain for a long time. On the other hand, vonoprazan is said to be stable even in acidic environments, and so can remain for a long time and exert sustained activity^11,12^. This may be one reason why vonoprazan alone was extracted as significantly associated with hypocalcaemia among the PPIs investigated in this study.

Steroids reduce calcium absorption in the intestine and kidney, and have been associated with osteoporosis as an adverse effect. In this study, among steroids, only dexamethasone was extracted as a significant factor. Dexamethasone is often used for an extended period as an antiemetic^13^ and for prevention of edema during chemotherapy^14^, and shows a longer duration of action than prednisolone. These may be the reasons why dexamethasone was extracted as a risk factor. Further verification is needed regarding this issue.

Low pre-treatment levels of serum calcium have been reported as a risk factor for hypocalcaemia^15^. Our result was also in agreement with those of previous studies. ROC analysis identified a cut-off value of 9.3 mg/dL (≥ grade 1). Clinicians should pay close attention to the onset of hypocalcaemia after administration of denosumab among patients with pre-treatment levels of serum calcium ≤ 9.3 mg/dL and should consider the administration of medications to prevent hypocalcaemia before denosumab administration.

In the current study, pre-treatment level of ALP was identified as a risk factor, and ROC analysis showed a cut-off value of ALP ≥ 457 U/L. Previous study discussed elevated bone-specific alkaline phosphatase levels may indicate potential calcium deposition in osteoid and undermineralised bone matrix, a phenomenon that can persist for weeks or months after osteoclast inhibition^10^. Our result is consistent with previous findings^9,10,16^. Clinicians thus need to know about the incidence and severity of hypocalcaemia, especially in patients with pre-treatment levels of ALP ≥ 457 U/L.

Pre-treatment level of haemoglobin was also identified as a risk factor. As a result of ROC analysis, the cut-off value for haemoglobin was ≤ 10.4 g/dL. Anaemia is also a risk factor for electrolyte abnormalities^17^. In addition, the low haemoglobin level was also influenced by the nutritional status as a patient background factor, suggesting that hypocalcaemia was also influenced by decreases in dietary calcium intake and absorption^18^. Care regarding the onset of hypocalcaemia is thus warranted in patients with anaemia before denosumab administration.

On the other hand, renal dysfunction before administration of denosumab has been reported as a risk factor for hypocalcaemia^19^, but was not extracted as a risk factor in this study. This may be because the number of patients with creatinine clearance of National Cancer Institute Common Terminology Criteria for Adverse Events (NCI‑CTCAE) version 5^20^ grade 3 or higher were a clear minority, representing only 9 of the 327 patients. NCI-CTCAE is a standard assessment tool of treatment-related adverse events.

Several limitations to the current study need to be considered. First, the retrospective nature of the study may have decreased the validity of the data obtained. Second, since this study was performed at a single institute, prospective multicentre studies are needed to confirm these results.

In conclusion, concomitant use of vonoprazan or dexamethasone, low calcium level before denosumab administration, and high ALP and low haemoglobin were identified as significant predictors for the development of hypocalcaemia in cancer patients with bone metastasis. However, our findings need to be confirmed in further studies. Nevertheless, these results may assist in developing strategies to improve the safety, efficacy, and QoL among patients receiving denosumab.

## Patients and methods

### Study period and participants

Between January 2016 and February 2020, this study retrospectively analyzed 348 advanced cancer patients who were receiving denosumab at our hospital. The Medical Ethics Review Committee of the Kyoto Prefectural University of Medicine approved this study (approval no. ERB-C-867-2). All procedures were performed in accordance with the ethical standards of the Kyoto Prefectural University of Medicine Institutional Medical Ethics Review Committee and the 1964 Declaration of Helsinki and its later amendments. No prospective studies with human participants or animals were performed by any of the authors for this article. Given the retrospective nature of this work, the need to obtain informed consent was waived for the individual participants included in the study, in accordance with the standards of the Kyoto Prefectural University of Medicine Institutional Medical Ethics Review Committee.

### Extraction of variables

For the regression analysis of factors associated with denosumab-induced hypocalcaemia, variables were extracted manually from medical charts. Evaluated variables included factors that could potentially impact the development of hypocalcaemia: demographic data (sex, age, height, weight, body mass index, and body surface area), number of cycles, concomitant medications (PPIs, steroids), cancer type, presence of comorbidity (diabetes mellitus), and laboratory test values (serum calcium, albumin, ALP, haemoglobin, creatinine, and estimated creatinine clearance). Creatinine clearance was estimated using the Cockcroft and Gault equation based on serum creatinine, sex, age, and weight. Clinical information was extracted before the first dose of denosumab. Concomitant medication was defined as administration of another drug for ≥ 2 weeks at the time of evaluation. The level of denosumab-induced hypocalcaemia based on an albumin-adjusted serum calcium concentration was evaluated by NCI‑CTCAE version 5. The evaluation time was the time of onset of hypocalcaemia (Grade 1 or higher) for patients who developed hypocalcaemia, and as the lowest calcium level within 1 month after final denosumab administration for patients who did not develop hypocalcaemia.

### Statistical analysis

Independent variables were analysed for multicollinearity (correlation coefficient |r| ≥ 0.7), since when correlations exist among the variables, this can lead to unreliable and unstable results of regression analyses. Independent variables were extracted based on correlation strength with the level of the denosumab-induced hypocalcaemia (dependent variable) or clinical significance. First, univariate ordered logistic regression analysis between the outcomes and each potential independent variable was performed. Subsequently, a multivariate ordered logistic regression model was constructed by employing the forward stepwise selection procedure with the potential candidate variables. The model used a variable entry criterion of 0.15 and a variable retention criterion of 0.1. Ordered logistic regression analysis was employed, because the level of hypocalcaemia was evaluated by a graded scale and multiple factors really associated as predictors for the development of denosumab-induced hypocalcaemia had to be analysed concurrently. Optimal cut off thresholds were determined using receiver operating characteristic curve (ROC) analysis^21^.

For all statistical analyses, values of *P* < 0.05 (2-tailed) were considered significant. All analyses were performed using JMP version 14.3.0. (SAS Institute, Cary, NC).
